# Distinct metabolic signatures of Alzheimer's and Parkinson's disease revealed through genetic overlap

**DOI:** 10.1016/j.ebiom.2026.106254

**Published:** 2026-04-10

**Authors:** Sara E. Stinson, Alexey A. Shadrin, Zillur Rahman, Linn Rødevand, Iris J. Broce, Geir Selbæk, Hreinn Stefansson, Jan Haavik, Nadine Parker, Elise Koch, Oleksandr Frei, Kevin S. O'Connell, Olav B. Smeland, Srdjan Djurovic, Anders M. Dale, Dennis van der Meer, Ole A. Andreassen

**Affiliations:** a Centre for Precision Psychiatry, Division of Mental Health and Addiction, Oslo University Hospital & Institute of Clinical Medicine, University of Oslo, Oslo, Norway; b K.G. Jebsen Centre for Neurodevelopmental Disorders, University of Oslo and Oslo University Hospital, Oslo, Norway; c Center for Multimodal Imaging and Genetics, J. Craig Venter Institute, La Jolla, CA, USA; d University of California San Diego, La Jolla, CA, USA; e Department of Geriatric Medicine, Oslo University Hospital, Oslo, Norway; f The Norwegian National Centre for Ageing and Health, Vestfold Hospital Trust, Tønsberg, Norway; g AMGEN deCODE Genetics, Reykjavik, Iceland; h Department of Biomedicine, Faculty of Medicine, University of Bergen, Bergen, Norway; i Division of Psychiatry, Haukeland University Hospital, Bergen, Norway; j Department of Medical Genetics, Oslo University Hospital & University of Oslo, Oslo, Norway

**Keywords:** Metabolomics, Proglucagon, Genome-wide association study, Cardiometabolic disease, Alzheimer's disease, Parkinson's disease

## Abstract

**Background:**

Metabolic dysfunction is a major risk factor for neurodegeneration, yet the genetic architecture linking systemic metabolism to Alzheimer's disease (AD) and Parkinson's disease (PD) remains unclear.

**Methods:**

We integrated genome-wide association data for 249 circulating metabolites and proglucagon with summary statistics for AD, PD, and cardiometabolic traits. Genetic correlations, polygenic overlap, causal relationships, and shared genetic loci were quantified using linkage disequilibrium score regression, high-definition likelihood, bivariate mixture modelling, Mendelian randomisation, and conjunctional false discovery rate analyses, followed by functional and tissue-specific enrichment analyses.

**Findings:**

AD displayed a metabolic-genetic profile aligned with body mass index, type 2 diabetes, coronary artery disease, and stroke, whereas PD exhibited largely opposing patterns (Spearman's r_s_ = −0.26). Mendelian randomization analyses supported causal effects of lipoprotein subclasses, glutamine, and proglucagon on AD risk, with opposite or null effects in PD. Shared loci between metabolites and AD were enriched for lipid metabolism and cholesterol transport, whereas PD-associated loci were enriched for mitochondrial function, vesicle trafficking, and stress-response signalling.

**Interpretation:**

AD and PD are shaped by fundamentally distinct metabolic-genetic architectures. Metabolically targeted interventions, particularly those modulating lipid, amino acid, and proglucagon pathways, may require disease-specific and genetically informed strategies for prevention and treatment of neurodegenerative diseases.

**Funding:**

10.13039/501100009708Novo Nordisk Foundation (NNF23OC0099658), Marie Skłodowska-Curie Actions (801133), the 10.13039/501100005416Research Council of Norway (334920, 351751, 296030, 324252, 324499, 326813), the 10.13039/100000002National Institutes of Health (U24DA041123, R01AG076838, U24DA055330, OT2HL161847, 5R01MH124839-02), 10.13039/501100004785NordForsk (164218), 10.13039/501100006095South-Eastern Norway Regional Health Authority (2020060), and the 10.13039/501100007601European Union’s Horizon 2020 (847776, 964874, 101057454).

**Research in context**

**Evidence before this study**

Metabolic dysfunction is increasingly recognised as a contributor to neurodegenerative disease, but the extent to which cardiometabolic and neurodegenerative disorders share common metabolic pathways remains unclear. We searched PubMed and Google Scholar from inception to Dec 15, 2025, using the terms (“neurodegenerative disease” OR “Alzheimer's disease” OR “Parkinson's disease”) AND (“cardiometabolic disease” OR “body mass index” OR “type 2 diabetes” OR “coronary artery disease” OR “stroke”) AND (“metabolomics” OR “metabolites” OR “proglucagon”) AND (“genetics” OR “causal inference”). Prior studies have linked individual metabolites to Alzheimer's or Parkinson's risk and suggest that GLP-1/GCGR agonism can improve cognition and alter metabolomic profiles in animal models of obesity and type 2 diabetes. However, no study has systematically evaluated the shared and distinct genetic architecture linking circulating metabolites and proglucagon-derived hormones to both cardiometabolic traits and major neurodegenerative diseases. In particular, whether Alzheimer's disease and Parkinson's disease share common metabolic–genetic pathways, or are influenced by fundamentally distinct metabolic mechanisms, remains unresolved.

**Added value of this study**

Using large-scale genome-wide association data, we provide a comprehensive evaluation of the genetic relationships between 249 circulating metabolites, proglucagon, cardiometabolic traits, and risk of Alzheimer's and Parkinson's disease. We identify disease-specific patterns of genetic correlation, polygenic overlap, and causal genetic associations, demonstrating that Alzheimer's disease and Parkinson's disease are characterised by markedly different metabolic–genetic architectures. Specific lipid subclasses, glutamine and proglucagon showed opposing genetic and directional associations with Alzheimer's disease versus Parkinson's disease. We further identify a previously unreported link between genetic regulation of proglucagon and the neuroprotective *APOE* ε2 variant. Functional analyses revealed enrichment of lipid metabolism and cholesterol transport pathways in Alzheimer's disease, whereas Parkinson's disease was associated with pathways related to mitochondrial function, vesicle trafficking, and cellular stress responses.

**Implications of all the available evidence**

These findings indicate that Alzheimer's disease and Parkinson's disease are not characterised by a shared metabolic signature of neurodegeneration, but instead by distinct metabolic perturbations. This distinction provides a biological framework for understanding why metabolically targeted interventions, including GLP-1–based therapies, may show heterogeneous or disease-specific effects. Collectively, the evidence supports a pathway-informed, disease-specific approach to biomarker development, risk stratification, and therapeutic targeting, and highlights lipid, amino acid, and proglucagon-related pathways as promising targets for precision prevention and treatment of neurodegenerative disease.

## Introduction

Neurodegenerative diseases are rising sharply with ageing populations, posing profound societal and economic challenges.^1^ Alzheimer's disease (AD) and Parkinson's disease (PD) are the most common neurodegenerative diseases,^2^ and both have been increasingly linked to systemic metabolic dysfunction.^3^

Cardiometabolic diseases, including type 2 diabetes (T2D), cardiovascular disease (CVD), and obesity are associated with accelerated cognitive decline and increased risk of dementia, with lifestyle factors such as physical inactivity, smoking, and excess alcohol intake further compounding risk.^4^ Epidemiological studies have consistently linked midlife obesity, hyperglycemia, and T2D with higher risk of AD,^5^, ^6^, ^7^ while T2D has been associated with faster disease progression in PD.^8^, ^9^, ^10^ However, genetic evidence for shared mechanisms between cardiometabolic and neurodegenerative diseases remains inconsistent.^11^ Some studies report overlapping genetic architecture between T2D and AD,^12^, ^13^, ^14^, ^15^ whereas others suggest limited or heterogeneous overlap, particularly for PD.^16^

One challenge in this field is that cardiometabolic risk factors represent broad clinical phenotypes that capture multiple underlying biological processes. Advances in high-throughput metabolomics now allow detailed characterisation of circulating lipids, lipoproteins, fatty acids, amino acids, and other metabolic intermediates in large population cohorts.^17^ These molecular phenotypes provide a more precise window into systemic regulation and its link to disease risk. Similarly, large-scale plasma proteomics enables the study of metabolically relevant hormones, including proglucagon-derived peptides.^18^

Proglucagon (GCG) is cleaved into several hormones, including glucagon and glucagon-like peptide-1 (GLP-1), which regulate glucose, lipid, and amino acid metabolism, exerting pleiotropic effects across peripheral tissues and in the brain.^19^^,^^20^ Incretin-based therapies, originally developed for T2D, induce substantial weight loss and improve cardiometabolic health, and growing evidence suggests potential neuroprotective effects.^21^ Preclinical studies^22^ and clinical trials^23^, ^24^, ^25^ indicate that GLP-1 receptor agonists (GLP1-RAs) may slow cognitive decline or modify disease progression in AD and PD, although results have been mixed.^26^ The biological basis for these differential effects remains unclear.

Despite increasing recognition of metabolic contributions to neurodegeneration, it remains unclear whether AD and PD share common metabolic–genetic pathways or whether they are influenced by fundamentally distinct metabolic architectures. Clarifying these relationships is essential for understanding disease mechanisms and for guiding metabolically targeted prevention and treatment strategies.

**Fig. 1:**
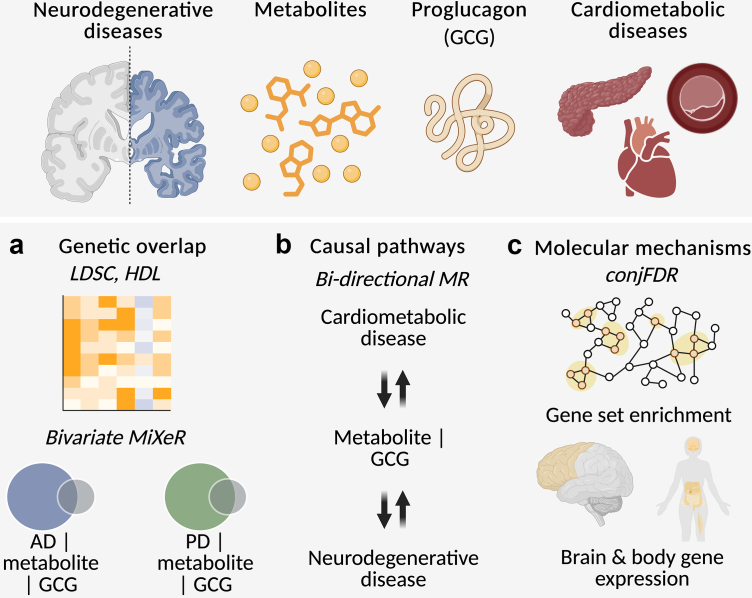
Graphical abstract. This study investigated the genetic overlap between circulating metabolites, proglucagon-derived hormones, cardiometabolic traits, and neurodegenerative diseases. (**a**) Global genetic overlap was estimated using LDSC, HDL, and bivariate MiXeR. (**b**) Bidirectional causal relationships were assessed using MR. (**c**) Shared genetic loci were identified through conjFDR, followed by gene set enrichment and tissue-specific gene expression analyses (body and brain) to investigate functional pathways and biological relevance. Figure created with Biorender.com. Abbreviations: CMD, cardiometabolic disease; conjFDR, conjunctional false discovery rate; GCG, proglucagon; HDL, high-definition likelihood; LDSC, linkage disequilibrium score regression; MiXeR, bivariate Gaussian mixture modelling; MR, Mendelian randomization; NDD, neurodegenerative disease.

In this study, we investigated the genetic overlap between circulating metabolites, proglucagon, cardiometabolic traits, and neurodegenerative diseases, with a focus on AD and PD. Using large-scale genome-wide association data, we quantified genetic correlations, polygenic overlap, causal pathways, and identified shared loci, followed by functional and tissue-specific enrichment (Fig. 1). We hypothesised that AD and PD would show distinct metabolic–genetic profiles, reflecting differences in how systemic metabolic dysregulation contributes to disease risk. This integrative approach aims to provide a mechanistic framework linking metabolic regulation to neurodegeneration and to inform disease-specific strategies for precision prevention and intervention.

## Methods

### Study population

The UK Biobank (application 27412) was utilised for this study. Our phenotypic analyses were restricted to unrelated individuals of White British ancestry identified via a KING kinship coefficient threshold of 0.05 and principal component analysis of ancestry.^27^ Participants were included if they had Nightingale NMR plasma metabolomics data and complete covariate information available (N = 207,841; mean age = 57.4 years, standard deviation [SD] = 8.0; 53.7% female). Sex was self-reported by study participants and recorded as a binary variable (male/female; UK Biobank field 31).

### Ethics

Ethical clearance was obtained from the NHS National Research Ethics Service (reference 11/NW/0382), and all participants provided informed consent, and is in accordance with the Declaration of Helsinki.

### Phenotypic associations

Metabolite measures were pre-processed with the ‘ukbnmr’ R package (version 4.4) to mitigate technical artifacts,^28^ followed by a rank-based inverse normal transformation (Supplementary Table S1).^29^ Diagnoses for AD (ICD-10 codes: F00, G30; n = 1746), PD (G20–G21; n = 1910), T2D (E11; n = 17,477), coronary artery disease (CAD) (I20–I25; n = 25,705), and stroke (I60–I64, G45; n = 8681) were extracted from electronic health record data (UK Biobank field 41270). Body mass index (BMI) (mean = 27.4, SD = 4.8; n = 207,841) was extracted from UK Biobank field 21001. Logistic regression was used for binary disease outcomes, and linear regression was used for BMI, adjusting for age and sex. Multiple testing was controlled for using the false discovery rate (FDR) method, with a threshold <5%.

### Genome-wide association study (GWAS) summary statistics

We incorporated GWAS summary statistics for 249 plasma metabolites derived from a recent meta-analysis performed by our group (Nightingale NMR platform, n = 300,486 UK Biobank and Estonian Biobank participants of European ancestry).^17^ For GCG, we used GWAS summary statistics from the affinity-based plasma proteomics dataset (Olink Explore 3072 platform, n = 34,557 UK Biobank individuals of European ancestry).^18^ GWAS summary statistics for AD,^30^ PD,^31^ T2D,^32^ CAD,^33^ stroke,^34^ and BMI^35^ were obtained from the latest large-scale European ancestry cohorts (Supplementary Table S2). AD and PD datasets excluding UK Biobank participants were utilised to avoid sample overlap.^36^ For sensitivity analysis, we obtained GWAS summary statistics for both AD and PD from the FinnGen release 12.^37^ Summary statistics were harmonised using the ‘cleansumstats’ pipeline (https://github.com/BioPsyk/cleansumstats).

### Linkage disequilibrium score regression (LDSC)

Cross-trait genetic correlations were estimated with LDSC (v1.0.0).^38^ Summary statistics were prepared according to LDSC requirements, including ‘munging’ and removal of variants located within the major histocompatibility complex (MHC) region (chromosome 6:25–35 Mb). Intercepts were freely estimated in case of potential residual confounding from sample overlap or stratification.

### High-definition likelihood (HDL)

To complement the LDSC analyses, cross-trait genetic correlations were performed using HDL (v1.4.2).^39^ GWAS summary statistics were harmonised using the same quality-control procedures for LDSC, including removing the MHC region. Default parameters were applied, and the imputed HapMap3 European reference panel was used.^39^

### Bivariate Gaussian mixture modelling (MiXeR)

To quantify polygenic overlap, we applied the bivariate MiXeR framework,^40^ which partitions variants into four components: null (π_0_), trait-specific (π_1_ and π_2_), and shared (π_12_) components. Model selection was based on the Akaike information criterion (AIC), which compares the best-fitting model to two reference scenarios: (i) a baseline infinitesimal model (minimum overlap) and (ii) a model assuming maximum possible overlap constrained by the least polygenic trait. The MHC region was excluded from all analyses, and the *APOE* region was removed for AD, to avoid inflation, which can bias estimates of polygenicity, as previously described.^11^

### Mendelian randomization (MR)

Bidirectional two-sample MR was conducted using the ‘TwoSampleMR’ R package v.0.6.14.^41^ The MHC region was excluded and sensitivity analysis without the *APOE* region was performed for AD. Variants were filtered for genome-wide significance and clumped for linkage disequilibrium (PLINK parameters: p < 5 × 10^−8^, r^2^ < 0.001, 10,000 kb window) using the 1000 Genomes European reference panel. Steiger filtering (p < 0.05) was applied to retain instrumental variables (IVs) which explained more variance (R^2^) in the exposure than in the outcome, thereby reducing the likelihood of reverse causation.^42^ Causal effects were estimated using Inverse Variance Weighted (IVW) regression, with the Weighted Median and MR-Egger methods applied to test robustness and detect pleiotropy.^43^ Associations were considered robust if they passed FDR correction (<5%) in IVW and Weighted Median, with nominal significance in MR-Egger (p < 0.05).

### Conjunctional false discovery rate (conjFDR)

Shared loci were identified via conjFDR analyses using the ‘pleioFDR’ software (https://github.com/precimed/pleiofdr),^44^ excluding the MHC region. Independent significant SNPs were defined as SNPs with conjFDR <0.05 that were independent at r^2^ < 0.6. SNPs in high LD (r^2^ > 0.6) with independent SNPs and with conjFDR <0.10 were designated candidate SNPs, and locus boundaries were defined as the span of these SNPs; loci within 250 kilobase (kb) were merged. Lead SNPs were selected as independent SNPs further independent at r^2^ < 0.1, with the locus lead variant defined as the SNP with the lowest conjFDR. LD r^2^ values were calculated using European-ancestry samples from the 1000 Genomes Project Phase 3 reference panel.^45^

### Gene mapping

Lead SNPs identified with conjFDR were mapped to genes via the variant-to-gene (V2G) algorithm from Open Targets Genetics.^46^ This approach integrates multiple sources of evidence, including quantitative trait loci data, chromatin interaction profiles, computational functional predictions, and proximity to transcription start sites.

### Gene set enrichment and tissue expression

For enrichment of the GCG-associated loci, we applied GSA-MiXeR,^47^ which accounts for LD and allows enrichment testing of small gene sets (<10 genes), to highlight biologically specific pathways. Gene sets with an FDR <5% were considered significant.

For metabolic marker-disease loci identified by conjFDR, enrichment was tested against Gene Ontology biological process terms from the Molecular Signatures Database (MSigDB v7.1, c5.bp). Gene expression enrichment was evaluated using RNA-seq data from GTEx v8 (https://gtexportal.org, 54 tissues) and normalised microarray profiles from the Allen Human Brain Atlas (https://human.brain-map.org/, multiple brain regions and adult donors). Enrichment analyses were performed in R using the ‘clusterProfiler’ Bioconductor R package v.4.12.6,^48^ with Fisher's exact tests. Significant GO terms were defined as those containing 5–500 mapped genes and a q-value <0.05. To reduce redundancy, if >80% of the genes in a smaller term were also present in a larger term, the overlapping terms were collapsed and the term with the lowest q-value was retained.

### Statistics

All data processing, harmonisation, and statistical analyses were performed using R statistical software, v.4.5.2.^49^ Power calculations were not applicable given the study design, but effective sample sizes for GWAS datasets are reported in Supplementary Table S2.

### Role of funders

The funders had no role in study design, data collection, data analysis, data interpretation, or writing of the report.

## Results

### Phenotypic analysis demonstrate that AD and PD metabolite profiles diverge from BMI associations

We first examined phenotypic associations between 249 plasma metabolites (228 lipids, lipoproteins, or fatty acids and 21 non-lipid traits) and major neurodegenerative diseases and cardiometabolic traits in 207,841 UK Biobank participants. Disease diagnoses were derived from ICD-10 codes for AD (n = 1746 cases), PD (n = 1910), T2D (n = 17,477), CAD (n = 25,705), and stroke (n = 8681). BMI (mean = 27.4, SD = 4.8) was analysed as a continuous trait. Associations were estimated using sex- and age-adjusted linear or logistic regression. Both AD and PD showed widespread associations with metabolites, with moderately correlated phenotypic patterns (AD-PD: Spearman's r_s_ = 0.58; Supplementary Table S3; Supplementary Fig. S1b). Notably, BMI demonstrated opposing relationships with both neurodegenerative diseases (BMI-PD: r_s_ = −0.32; BMI-AD: r_s_ = −0.18).

### Genetic overlap reveals distinct metabolic profiles in AD and PD

We next examined the genetic overlap between the circulating metabolites, proglucagon, neurodegenerative diseases, and cardiometabolic traits, using LDSC, HDL, and bivariate MiXeR.

**Fig. 2:**
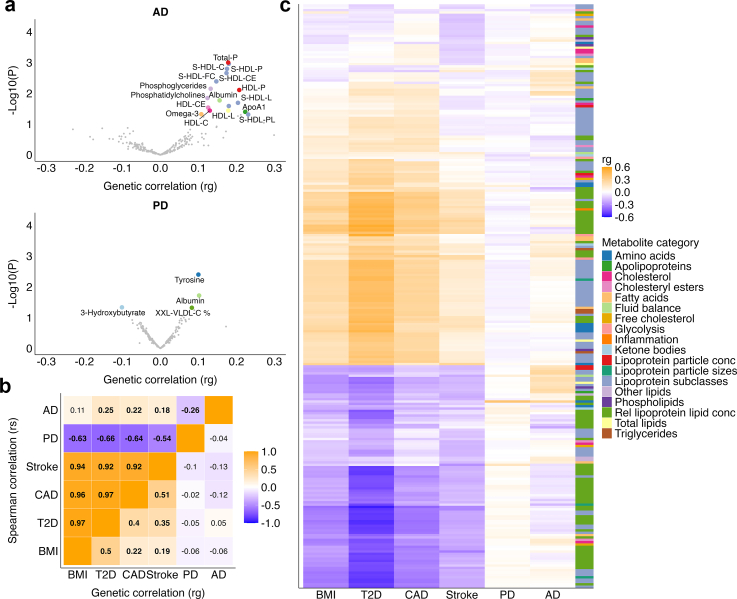
Genetic correlations (LDSC) between circulating metabolites, cardiometabolic traits, and neurodegenerative diseases. (**a**) Volcano plots of genetic correlations (x-axis) and -log10(P) (y-axis) for AD and PD. Metabolites with nominally significant correlations (P ≤ 0.05 [LDSC]) are coloured by metabolic category and annotated; non-significant metabolites (P > 0.05 [LDSC]) are shown in grey. (**b**) Correlation matrix of pairwise genetic correlation (r_g_, lower right triangle) and Spearman correlation coefficients (r_s_) of the LDSC estimates across all 249 metabolites (upper left triangle). Significant correlations (FDR <0.05 [LDSC]) are shown in bold. (**c**) Heatmap of genetic correlations (r_g_) between metabolites (rows), cardiometabolic traits (BMI, T2D, CAD, and stroke), and neurodegenerative diseases (AD and PD) (columns). Metabolites were hierarchically clustered, with metabolite categories indicated in the adjacent colour bar. Abbreviations: AD, Alzheimer's disease; BMI, body mass index; CAD, coronary artery disease; CMD, cardiometabolic disease; FDR, false discovery rate; LDSC, linkage disequilibrium score regression; PD, Parkinson's disease; r_g_, genetic correlation; T2D, type 2 diabetes.

Across the 249 metabolites, genetic correlation estimates differed markedly between AD and PD (Fig. 2a). While the phenotypic pattern of associations with metabolites and neurodegenerative diseases were moderately correlated (AD-PD: r_s_ = 0.58), their genetic pattern of associations with metabolites were discordant (AD-PD: r_s_ = −0.26; Fig. 2b; Supplementary Table S4). Notably, the direct pairwise genetic correlation between AD and PD was not statistically significant (r_g_ = −0.04, p = 0.59 [LDSC]; Fig. 2b; Supplementary Table S5).

Overall, AD showed a significant positive pattern of genetic correlations with metabolites, similar to cardiometabolic traits (BMI: r_s_ = 0.11; T2D: r_s_ = 0.25; CAD: r_s_ = 0.22; and stroke: r_s_ = 0.18; Fig. 2b). In contrast, PD exhibited an inverse pattern of genetic correlations with metabolites, opposite to cardiometabolic traits (BMI: r_s_ = −0.63; T2D: r_s_ = −0.66; CAD: r_s_ = −0.64; and stroke: r_s_ = −0.54; Fig. 2b). This divergence was observed across lipid subclasses, amino acids, and other metabolic categories (Fig. 2c).

Sensitivity analyses using FinnGen data demonstrated a highly consistent pattern (Supplementary Table S4). Similarly, AD showed positive patterns of genetic correlations with metabolites, in line with cardiometabolic traits (BMI: r_s_ = 0.29; T2D: r_s_ = 0.42; CAD: r_s_ = 0.37; stroke: r_s_ = 0.42), whereas PD exhibited inverse correlations (BMI: r_s_ = −0.66; T2D: r_s_ = −0.72; CAD: r_s_ = −0.74; stroke: r_s_ = −0.59; Supplementary Fig. S2).

To further validate our LDSC findings, we performed cross-trait genetic correlation analyses using HDL. Overall, the patterns of HDL estimates were concordant in both direction and relative magnitude (Supplementary Table S6). AD demonstrated predominantly positive genetic correlations with circulating metabolites, similar to cardiometabolic traits, whereas PD showed inverse correlations across the cardiometabolic trait spectrum.

Proglucagon (GCG) (Supplementary Fig. S3) demonstrated a significant positive genetic correlation with T2D (r_g_ = 0.21; p = 0.001; FDR <0.05 [LDSC]), but not with AD (r_g_ = 0.18; p = 0.18 [LDSC]) or PD (r_g_ = −0.06; p = 0.45 [LDSC]; Supplementary Fig. S4a; Supplementary Table S7). Sensitivity analyses in FinnGen demonstrated consistent direction of effects for GCG, with a positive genetic correlation for AD (rg = 0.43; p = 0.048 [LDSC]) and an inverse genetic correlation for PD (rg = −0.30; p = 0.005 [LDSC]; Supplementary Table S7).

**Fig. 3:**
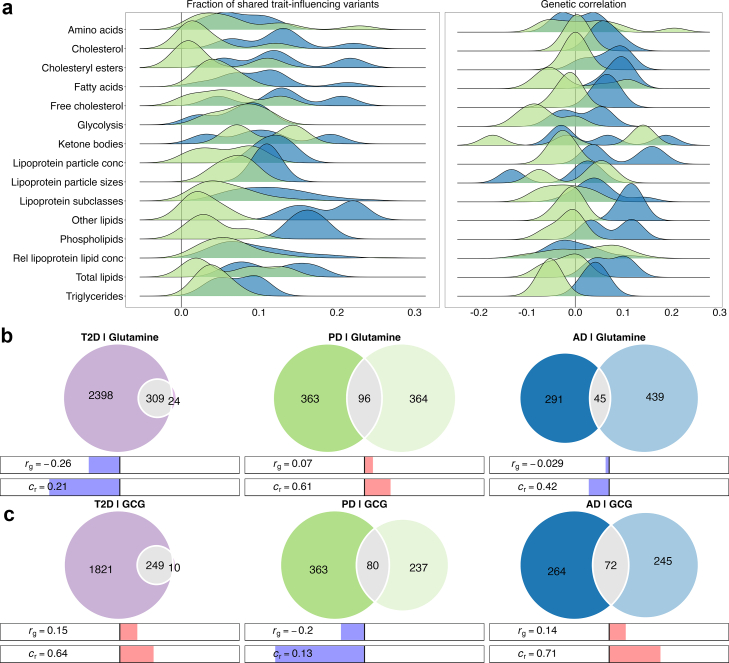
Genetic overlap (bivariate MiXeR) between circulating metabolic markers, cardiometabolic traits, and neurodegenerative diseases. (**a**) Distributions of the fraction of shared trait-influencing variants (left) and genetic correlations (right) across metabolite categories estimated using bivariate MiXeR. (**b and c**), Venn diagrams showing the number of unique and shared variants between glutamine, GCG, T2D, PD, and AD. The bars below the diagrams indicate genetic correlations (r_g_) and concordance rates (c_r_), where c_r_ = 0.50 indicates equal proportions of concordant and opposing effects. Abbreviations: AD, Alzheimer's disease; c_r_, concordance rate; GCG, proglucagon; MiXeR, bivariate Gaussian mixture modelling; PD, Parkinson's disease; r_g_, genetic correlation; T2D, type 2 diabetes.

To quantify polygenic overlap independent of effect direction, we applied bivariate MiXeR modelling. AD shared a larger fraction of trait-influencing variants with circulating metabolites than PD (median shared fraction 10.5% versus 3.1%; p = 1.81 × 10^−27^ [Wilcoxon rank-sum test]; Supplementary Table S8). Lipid-related metabolites accounted for the majority of shared variants with AD, whereas amino acids, ketone bodies, and fluid balance measures accounted for a larger proportion of shared variants with PD (Fig. 3a). Model fit improved over the baseline infinitesimal model for 236 of 249 metabolites with AD and 214 with PD.

For individual metabolites, patterns of genetic overlap differed between diseases. For glutamine, bivariate MiXeR estimated opposite directions of genetic correlation for AD (r_g_ = −0.03) and PD (r_g_ = 0.07; Fig. 3b; Supplementary Fig. S5). A similar pattern was observed for GCG, which showed positive genetic correlation with AD (r_g_ = 0.14) and negative correlation with PD (r_g_ = −0.20; Fig. 3c; Supplementary Fig. S6; Supplementary Table S9).

### Causal pathways between metabolic markers and neurodegenerative diseases

**Fig. 4:**
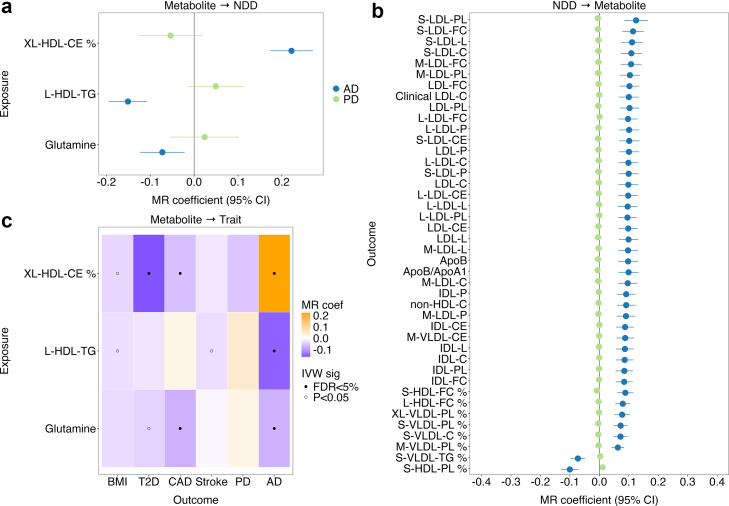
Bidirectional causal relationships between circulating metabolites, cardiometabolic traits, and neurodegenerative diseases. (**a**) Forest plot of MR estimates showing causal effects of metabolites on AD and PD, using IVW regression with 95% CIs. (**b**) Forest plot of MR estimates of causal effects of AD and PD on the top metabolites (IVW, FDR <1.0 × 10^−7^ [MR]). (**c**) Heatmap summarising significant bidirectional causal effects of metabolites on neurodegenerative diseases, and their effects on cardiometabolic traits. Abbreviations: AD, Alzheimer's disease; BMI, body mass index; CAD, coronary artery disease; CIs, confidence intervals; CMD, cardiometabolic disease; FDR, false discovery rate; IVW, inverse variance weighted; MR, Mendelian randomization; NDD, neurodegenerative disease; PD, Parkinson's disease; T2D, type 2 diabetes.

We assessed directional genetic relationships between circulating metabolites, proglucagon, cardiometabolic traits, and neurodegenerative diseases using bidirectional two-sample Mendelian randomization (Fig. 4 and Supplementary Tables S10–S12).

For AD, three metabolites showed robust evidence of causal effects: higher XL-HDL-CE %, lower L-HDL-TG, and lower glutamine, with consistent support across IVW, weighted median, and MR-Egger models following Steiger filtering (Fig. 4a). Sensitivity analysis in FinnGen confirmed concordant effect directions for all three metabolites. Robust replication across all MR methods was observed for L-HDL-TG and XL-HDL-CE %, whereas glutamine remained supported in the IVW model only (Supplementary Table S10). Given the established pleiotropic role of *APOE* in AD risk, we repeated the analyses excluding variants within this region. Effect directions were preserved for all three metabolites; however, the association for L-HDL-TG was attenuated, suggesting partial dependence on *APOE* (Supplementary Table S10).

In contrast, no metabolites demonstrated robust evidence of causal effects on PD (Fig. 4a). Although total-PL showed nominal significance in the MR-Egger model, this association was not supported by the IVW and weighted median models (Supplementary Fig. S7) and did not replicate in FinnGen (Supplementary Fig. S7).

In the reverse direction, genetic liability to AD showed directional associations with 172 circulating metabolites, predominantly involving lipid and lipoprotein subclasses, including cholesterol fractions and apolipoproteins (Fig. 4b; Supplementary Fig. S9). No metabolites showed robust directional associations with PD liability after multiple testing correction (Fig. 4b).

Several causal metabolites for AD exhibited disease-specific directional associations. For example, genetically lower circulating glutamine levels were associated with higher risk for CAD and AD (Fig. 4c; Supplementary Table S10). Likewise, higher XL-HDL-CE % was associated with lower risk of T2D and CAD, but higher risk of AD.

For proglucagon, T2D showed causal effects on circulating GCG levels (IVW FDR = 0.002; weighted median FDR = 0.002; MR-Egger p = 0.020 [MR]). In the reverse direction, GCG showed nominal evidence of association with AD risk (IVW p = 0.004, weighted median p = 0.015, MR-Egger: p = 0.868 [MR]), but not with PD (p > 0.05 all [MR]; Supplementary Fig. S4b and c; Supplementary Table S11).

We did not observe robust causal effects of BMI, T2D, CAD, or stroke on AD or PD risk, following multiple testing corrections (Supplementary Table S12). However, these cardiometabolic traits showed extensive associations with circulating metabolites, indicating that metabolic intermediates may represent downstream correlates of cardiometabolic genetic liability.

### Genetic regulation of proglucagon highlights apolipoprotein-driven lipid pathways

To define molecular pathways underlying the genetic regulation of GCG, we applied GSA-MiXeR,^47^ which revealed significant enrichment of lipid-related processes, including phosphatidylcholine-sterol O-acyltransferase activator activity (6 genes, FDR = 0.014), chylomicron biology (13 genes, FDR = 0.017), and triglyceride rich plasma lipoprotein particle (19 genes, FDR = 0.025; Supplementary Table S13). These enrichments were driven by *APOE*, *APOC1*, *APOA5*, and *APOA1*, uncovering a link between apolipoproteins and GCG signalling.

### Shared genetic loci between metabolic markers and neurodegenerative disorders

**Fig. 5:**
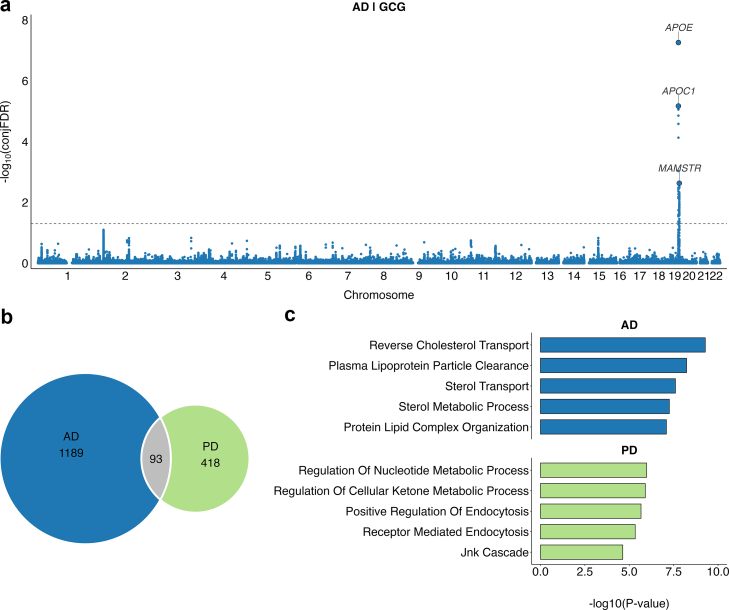
Shared loci and enriched pathways between circulating metabolic markers and neurodegenerative diseases. (**a**) conjFDR Manhattan plot for AD and GCG, highlighting shared loci on chromosome 19, *APOE* locus. (**b**) Venn diagram showing the number of unique and shared genes mapped from conjFDR loci across 249 metabolites for AD and PD. (**c**) Bar plots of top enriched GO biological process terms for conjFDR loci across 249 metabolites for AD and PD, showing -log10(p-values) on the x-axis [Fisher's exact test]. Abbreviations: AD, Alzheimer's disease; conjFDR, conjunctional false discovery rate; GCG, proglucagon; GO, Gene Ontology; PD, Parkinson's disease.

To identify shared genetic loci between circulating metabolites and neurodegenerative diseases, we performed conjFDR analyses across 249 metabolites and AD and PD. Across all metabolites, conjFDR identified 1282 variants jointly associated with metabolites and AD, and 511 variants jointly associated with metabolites and PD at conjFDR <0.05 (Fig. 5b; Supplementary Tables S14 and S15). Of these, 93 variants were common between AD and PD. Lead variants were mapped to genes using the Open Targets Genetics variant-to-gene framework.

For glutamine, conjFDR analyses identified 14 shared loci with AD and 62 shared loci with PD (Supplementary Figs. S10 and S11). AD-associated glutamine loci included rs2840676 in *LEPR*, previously linked to BMI, immune traits, and psychiatric disorders.^50^ PD-associated glutamine loci included rs11223625 (*IGSF9B*), rs11614702 (*FBRSL1*), and rs2274693 (*PTCH1*), which have been reported in association with anthropometric traits,^51^ glucose metabolism,^52^ and brain imaging phenotypes.^53^

For proglucagon, conjFDR identified three shared loci with AD on chromosome 19, mapping to *APOE*, *APOC1*, and *MAMSTR* (Fig. 5a; Supplementary Table S16). The lead variant rs7412, defining the *APOE* ε2 isoform, was among the shared loci.^54^ For PD, two GCG-associated loci were identified on chromosome 2, mapping to *EIF2B4* and *CCNT2-AS1*. The PD-associated lead variant rs80051818 in *EIF2B4* has previously been associated with circulating lipid traits.^55^

### Pathway enrichment for metabolites and neurodegenerative diseases

Shared variants between neurodegenerative diseases and metabolites were mapped to genes via Open Targets Genetics^46^ and tested for enrichment in Gene Ontology (GO) biological processes (Fig. 5c, Supplementary Tables S17 and 18). In AD, enriched pathways were strongly related to lipid and cholesterol biology, including reverse cholesterol transport, plasma lipoprotein particle clearance, sterol transport and metabolism, and protein–lipid complex organisation, highlighting the central role of lipid dysregulation in AD pathophysiology. In contrast, PD was characterised by enrichment of pathways involved in cellular energy metabolism (nucleotide and ketone processes), vesicle trafficking (positive and receptor-mediated endocytosis), and stress-response signalling via the JNK cascade.

### Tissue-specific expression of genes shared between metabolites and neurodegenerative diseases

To evaluate tissue specificity of genes mapped from conjFDR loci, we examined expression enrichment across brain regions and peripheral tissues using data from the Allen Human Brain Atlas and GTEx v8.

**Fig. 6:**
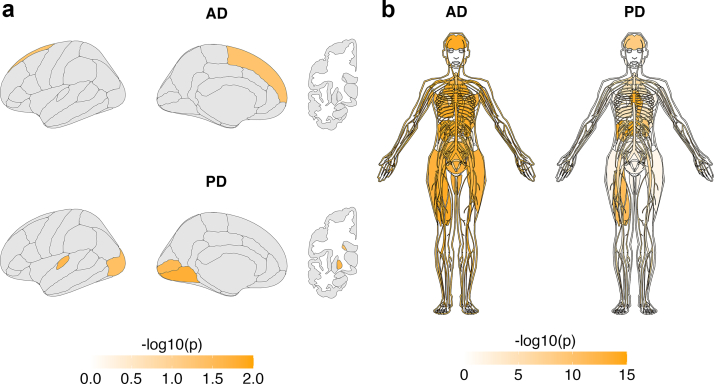
Tissue-specific enrichment of loci shared between circulating metabolites and neurodegenerative diseases. (**a**) Brain maps showing enrichment of shared genes across cortical (Desikan–Killiany atlas) and subcortical (Freesurfer aseg) regions using expression data from the Allen Human Brain Atlas. (**b**) Anatomograms showing enrichment of shared genes across 30 body tissues from GTEx v8. Regions or tissues with significant enrichment (p < 0.05 [Fisher's exact test]) are highlighted in orange. Abbreviations: AD, Alzheimer's disease, GTEx, Genotype-Tissue Expression project, PD, Parkinson's disease.

Within the brain, genes shared between metabolites and AD showed nominal enrichment in the superior frontal cortex (p = 0.048 [Fisher's exact test]), although this did not remain significant after FDR correction for multiple testing (Fig. 6a). Genes shared between metabolites and PD showed nominal enrichment across multiple cortical regions (Fisher's exact test), including the cuneus (p = 0.010), lingual gyrus (p = 0.017), transverse temporal cortex (p = 0.026), pericalcarine cortex (p = 0.028), and lateral occipital cortex (p = 0.039), as well as in subcortical structures including the pallidum (p = 0.025) and caudate nucleus (p = 0.046). None of the regional brain enrichments remained significant after FDR correction.

Across peripheral tissues, AD-associated metabolite genes showed significant enrichment (Fisher's exact test) in the brain (FDR = 1.07 × 10^−26^) and in multiple non-central nervous system tissues, including pancreas (FDR = 2.30 × 10^−27^), liver (FDR = 2.30 × 10^−27^), and heart (FDR = 3.70 × 10^−26^) (Fig. 6b). PD-associated metabolite genes were significantly enriched (Fisher's exact test) in the brain (FDR = 5.55 × 10^−9^) and in peripheral tissues including heart (FDR = 1.44 × 10^−22^), liver (FDR = 2.26 × 10^−21^), aorta (FDR = 4.28 × 10^−12^), and skeletal muscle (FDR = 1.64 × 10^−11^). The distribution of enriched tissues differed between AD and PD, with broader systemic enrichment observed for AD-associated metabolite genes.

## Discussion

In this large-scale integrative phenotypic and genetic study, we show that the metabolic pathways linking systemic metabolism to neurodegeneration are strongly disease specific. Although AD and PD exhibited positively correlated phenotypic associations with circulating metabolites, their genetic correlations were opposite in direction, indicating that similar downstream metabolic patterns may emerge despite contrasting genetic architectures. This divergence between phenotypic and genetic relationships is consistent with a potential contribution of shared environmental influences, disease-stage effects, or gene–environment interplay shaping the observed metabolic profiles.^56^ Together, these findings underscore the importance of distinguishing genetically driven metabolic liability from secondary metabolic changes occurring during disease progression. AD also displayed metabolic–genetic patterns aligned with cardiometabolic traits, whereas PD showed largely opposing or null patterns. These differences were consistent across multiple analytic frameworks, including genetic correlations, polygenic overlap, bidirectional MR analyses, conjFDR, and functional annotation, indicating that AD and PD are shaped by fundamentally distinct metabolic-genetic architectures.

Epidemiological evidence indicates that a higher midlife BMI increases dementia risk, whereas higher late-life BMI may be protective.^6^ Our phenotypic and genetic findings support the hypothesis that age-dependent metabolic shifts contribute to disease risk across the lifespan.^57^ At a genome-wide level, AD shared a substantially greater fraction of trait-influencing variants with circulating metabolites than PD, with lipids and lipoprotein subclasses dominating this overlap. In contrast, PD showed more limited overlap, primarily involving amino acids, ketone bodies, and energy-related metabolites. These findings extend previous reports of limited direct genetic overlap between AD and PD^36^ by demonstrating that, even when overlap exists, the metabolic context in which genetic risk operates differs profoundly between the two neurodegenerative diseases.

MR analyses further supported this distinction. For AD, we identified causal evidence for higher XL-HDL-CE %, alongside lower L-HDL-TG and glutamine levels, with extensive reverse effects of AD liability on circulating lipid profiles. These bidirectional relationships suggest a complex interplay in which metabolic dysregulation both contributes to, and is exacerbated by, neurodegenerative processes in AD. In contrast, PD showed little evidence of robust directional relationships with circulating metabolites, consistent with a weaker coupling between systemic metabolic regulation and disease risk.

The causal signal for XL-HDL-CE % points to a role for disrupted reverse cholesterol transport in AD pathogenesis. During reverse cholesterol transport, HDL acquires free cholesterol from peripheral tissues, which is then esterified into cholesteryl esters (CEs) by lecithin:cholesterol acyltransferase (LCAT), driving maturation into extra-large CE-enriched HDL particles.^58^ More broadly, pharmacological reduction of CE biosynthesis supports a pathogenic role for this pathway: ACAT inhibitors attenuate amyloid pathology in AD mouse models.^59^

For L-HDL-TG, the causal direction, whereby lower triglyceride content within large HDL particles increases AD risk, is consistent with recent large-scale epidemiological data demonstrating independent associations of low HDL-C and low triglyceride levels with AD risk.^60^

Genetically lower circulating glutamine levels were associated with increased AD risk, in line with recent MR findings,^61^ whereas PD trended in the opposite direction. As a key precursor for glutamate, glutamine plays a central role in synaptic transmission and NMDA receptor signalling, pathways implicated in AD pathophysiology.^62^ Glutamine metabolism is also closely linked to insulin sensitivity and body weight regulation,^63^ providing a plausible mechanistic bridge between cardiometabolic dysfunction and AD. The opposing pattern observed in PD highlights that metabolic perturbations may not exert uniform effects across neurodegenerative diseases.

Narrowing conjFDR results to metabolites with causal effects, highlighted loci such as rs2840676 in *LEPR* for AD-glutamine, linking leptin signalling, inflammation, and energy homoeostasis.^50^^,^^64^ PD-glutamine loci include *IGSF9B, FBRSL1*, and *PTCH1*, which are associated with body composition,^51^ glucose metabolism,^52^ and brain structure.^53^

Our analyses also implicate proglucagon signalling as a potential interface between cardiometabolic disease and AD. Genetic evidence supported an effect of T2D liability on circulating proglucagon levels, alongside suggestive directional effects of proglucagon on AD risk. Functional enrichment analyses indicated that genetic regulation of proglucagon is closely linked to apolipoprotein-mediated lipid pathways, including *APOE*, *APOC1*, and *APOA1*. Notably, shared loci between proglucagon and AD included rs7412, which defines the neuroprotective *APOE* ε2 isoform,^54^ highlighting a convergence between lipid metabolism, proglucagon signalling, and AD susceptibility. These findings provide a genetic framework that may help explain heterogeneous responses to incretin-based therapies observed across neurodegenerative diseases and patient subgroups.^26^

Functional annotation of shared metabolic loci further underscored distinct disease mechanisms. In AD, metabolite-associated loci were enriched for pathways involved in lipid metabolism, cholesterol transport, and lipoprotein particle remodelling, processes central to amyloid-β clearance, neuronal membrane integrity, and APOE function.^65^ In PD, enriched pathways were related to mitochondrial energy metabolism, vesicle trafficking, and stress-response signalling, consistent with impaired bioenergetics, disrupted protein handling, and selective vulnerability of dopaminergic neurons.^66^ Only a small number of loci were shared between AD and PD, reinforcing the view that their metabolic–genetic links are largely distinct.

Tissue-expression analyses provided additional context. Genes linking metabolites to AD showed enrichment not only in the brain but also across peripheral tissues central to metabolic regulation, including pancreas, liver, and heart, supporting a model in which chronic systemic metabolic dysfunction contributes to cerebral vulnerability.^67^ PD-associated metabolic genes were enriched in multiple brain regions relevant to motor and cognitive function, as well as in peripheral tissues involved in energy metabolism and autonomic regulation.^68^ These patterns suggest that, while both disorders involve brain-expressed genes, AD may be more strongly influenced by systemic metabolic perturbations, whereas PD may be driven to a greater extent by cell-intrinsic neuronal processes.

Clinically, these findings have important implications. They challenge the assumption of a shared metabolic signature across neurodegenerative diseases and suggest that metabolically targeted interventions are unlikely to have uniform effects. Instead, therapies aimed at improving lipid handling, amino acid metabolism, or proglucagon signalling may be more relevant for AD than for PD and may require genetic or metabolic stratification to maximise benefit. More broadly, our results support a precision medicine framework in which metabolic biomarkers are used to guide disease-specific prevention and intervention strategies.

Strengths of this study include the integration of large-scale metabolomics,^17^ proteomics,^18^ and genetic data, enabling a comprehensive assessment of metabolic–genetic relationships across cardiometabolic and neurodegenerative diseases. This cross-disease design allows identification of both shared and disease-specific metabolic signatures. Limitations include the restriction to individuals of European ancestry, which may limit generalisability. Another limitation includes the inability to distinguish individual proglucagon-derived peptides with certainty using affinity-based proteomics. Nevertheless, prior experimental work supports that the assay captures biologically relevant combination of intestinal and pancreatic proglucagon products (most likely GLP-1 and major proglucagon fragment).^69^

In conclusion, our findings demonstrate that AD and PD are characterised by distinct metabolic–genetic architectures rather than a shared metabolic basis for neurodegeneration. Lipid- and apolipoprotein-related pathways, alongside amino acid and proglucagon signalling -emerge as central mechanisms—linking cardiometabolic dysfunction to AD, whereas PD is more strongly associated with pathways related to mitochondrial function, vesicle trafficking, and cellular stress responses. These insights provide a mechanistic rationale for disease-specific metabolic interventions and underscore the importance of genetically informed approaches to the prevention and treatment of neurodegenerative disease.

## Contributors

S.E.S., A.A.S., Z.R., S.D., A.M.D., D.v.d.M., and O.A.A. contributed to conceptualisation of the study. Data curation was performed by S.E.S., A.A.S., Z.R., N.P., O.F., O.B.S., and D.v.d.M. Formal analysis was conducted by S.E.S., A.A.S., Z.R., E.K., and D.v.d.M. Funding acquisition was secured by S.E.S., K.S.O., A.M.D., D.v.d.M., and O.A.A. Methodology was developed by S.E.S., A.A.S., Z.R., N.P., O.F., K.S.O., O.B.S., and D.v.d.M. Project administration was carried out by G.S., H.S., J.H., S.D., A.M.D., D.v.d.M., and O.A.A. Software development was undertaken by A.A.S., Z.R., N.P., O.F., K.S.O., O.B.S., A.M.D., D.v.d.M., and O.A.A. Supervision was provided by D.v.d.M. and O.A.A. Validation was performed by S.E.S. and A.A.S. Visualisation was conducted by S.E.S. and D.v.d.M. The original draft of the manuscript was written by S.E.S., D.v.d.M., and O.A.A. All authors contributed to review and editing of the manuscript. S.E.S. and D.v.d.M. accessed and verified the underlying data. All authors read and approved the final version of the manuscript.

## Data sharing statement

Access to individual-level data is available through application to the UK Biobank (www.ukbiobank.ac.uk), contingent upon approval from the UK Biobank Access Committee, and analyses must be conducted on the UK Biobank Research Analysis Platform (UKB-RAP). GWAS summary statistics for the Nightingale NMR metabolomics data were previously deposited in Zenodo (https://zenodo.org/records/15420219).^17^ GWAS summary statistics for proglucagon (Olink proteomics) are available through Sun et al. (2023).^18^ Publicly available GWAS summary statistics for AD, PD, T2D, CAD, stroke, and body mass index were obtained from published resources.^30^, ^31^, ^32^, ^33^, ^34^, ^35^ The code used for data analysis is available on GitHub: https://github.com/sarastinson/metabo_ndd.^70^

## Declaration of interests

G.S. has received honoraria for lectures and symposia sponsored by Eisai and Eli Lilly, and has served on Advisory Boards for Roche, Eli-Lilly, and Eisai in relation to disease-modifying therapies for Alzheimer's disease. O.F. is a consultant to Precision Health. A.M.D. is the founding director, holds equity in CorTechs Labs, Inc. (DBA Cortechs.ai), and serves on its Board of Directors. A.M.D. is also the President of J. Craig Venter Institute (JCVI) and is a member of the Board of Trustees of JCVI. A.M.D. is an unpaid consultant for Oslo University Hospital. O.A.A. has received speaker fees from Lundbeck, Janssen, Otsuka, and Sunovion, and serves as a consultant to Cortechs.ai and Precision Health. All the other authors report no competing interests.
