# Supplementary material for: Distinct metabolic signatures of Alzheimer's and Parkinson's disease revealed through genetic overlap
Source: eBioMedicine. 2026 Apr 10;127:106254. doi: 10.1016/j.ebiom.2026.106254 (PMC13091360; doi:10.1016/j.ebiom.2026.106254)
Supplement: Supplementary Figures [file mmc1.docx]

**Supplementary Fig. S1**

**
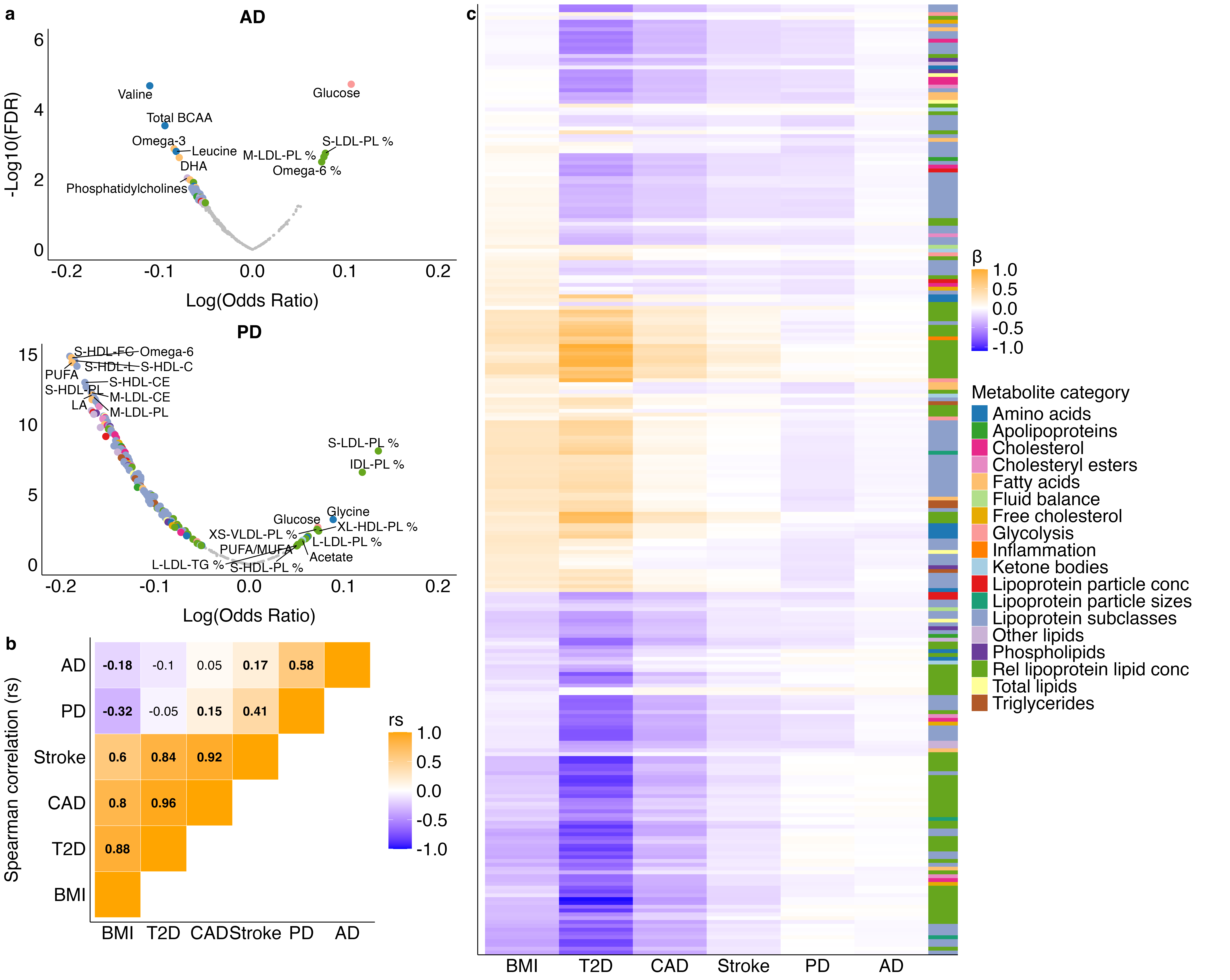
**

**Supplementary Fig. S2**


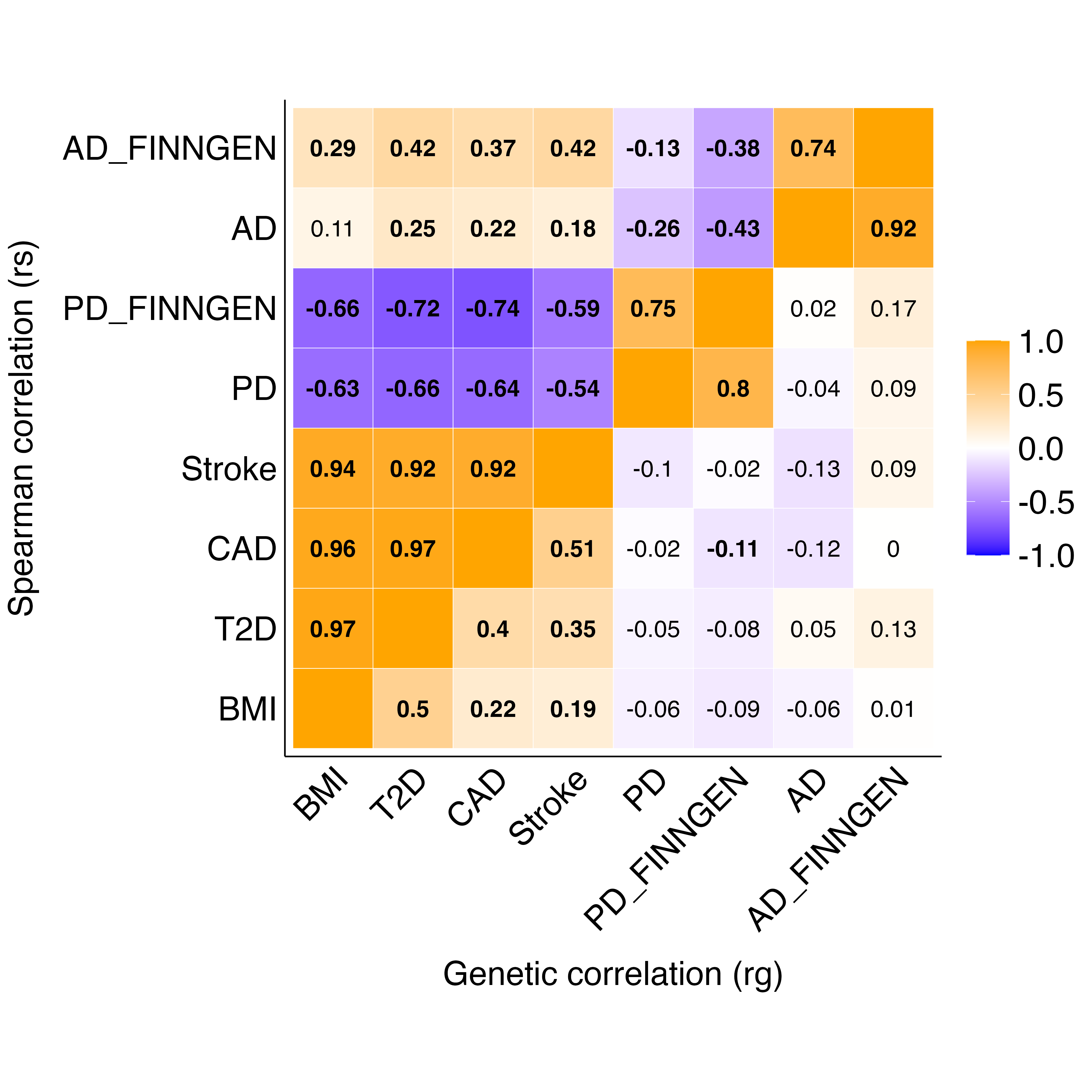
**Supplementary Fig. S3**

***
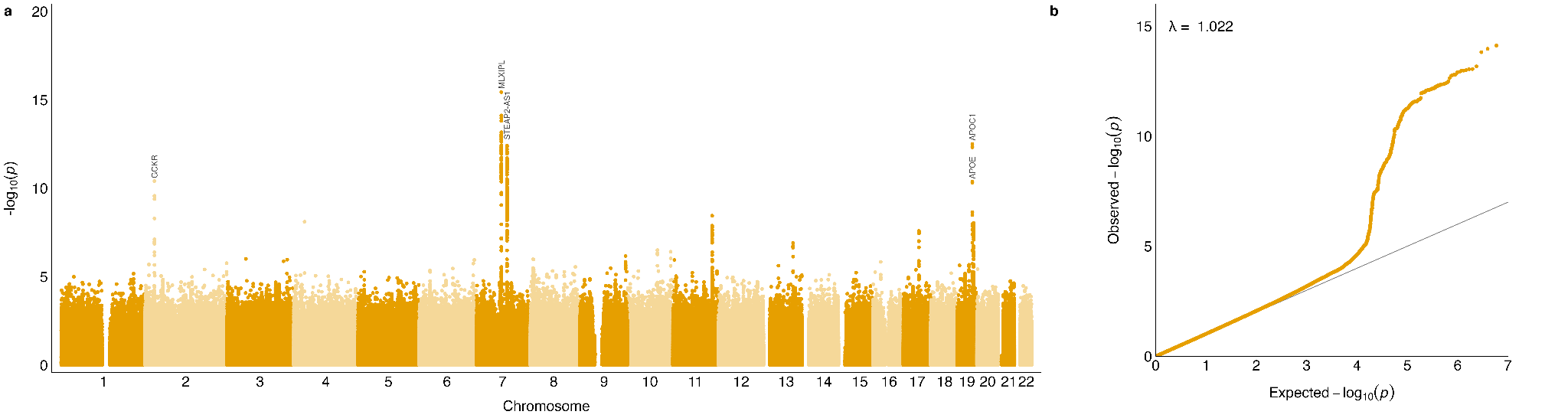
***

**Supplementary Fig. S4**


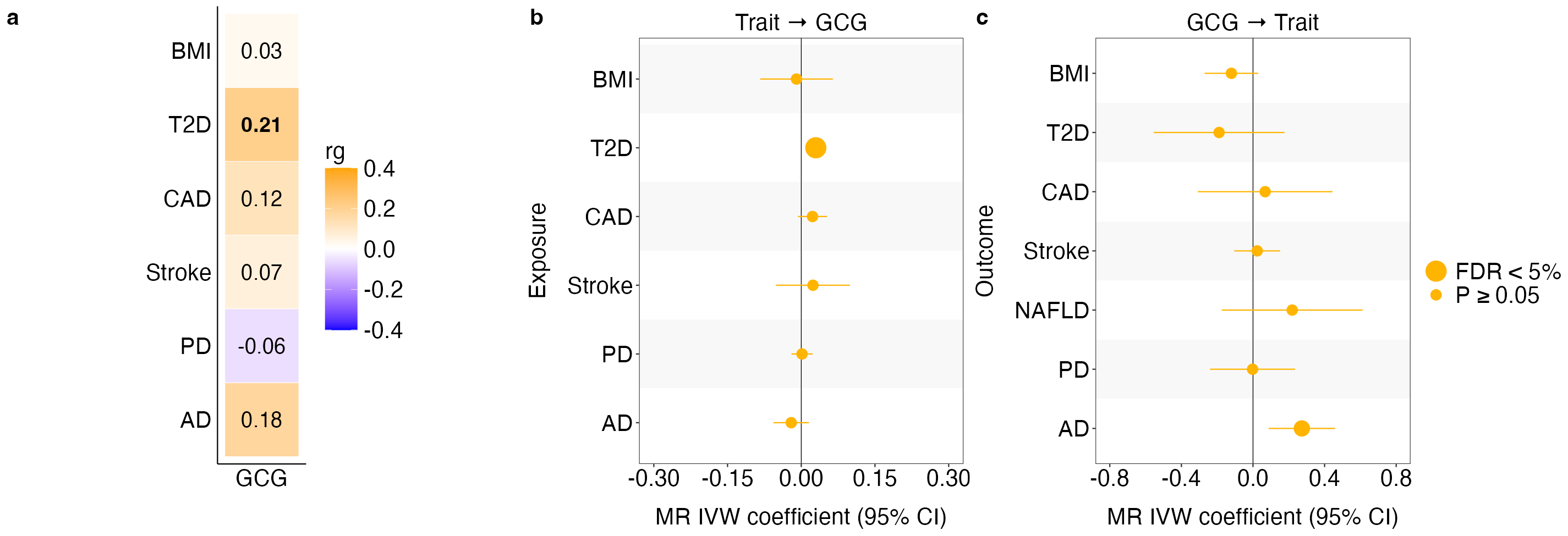


**Supplementary Fig. S5**

*
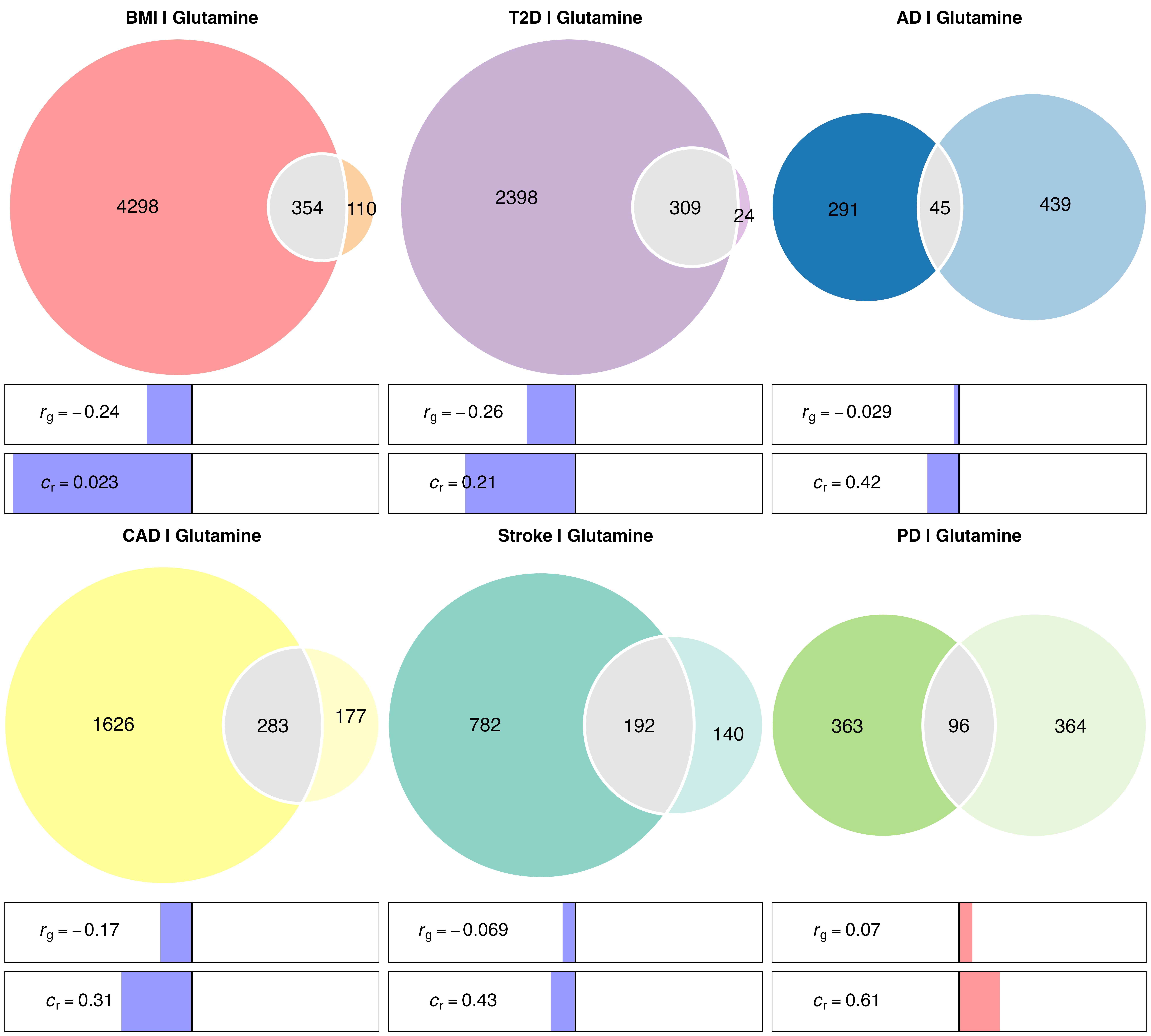
*

**Supplementary Fig. S6**


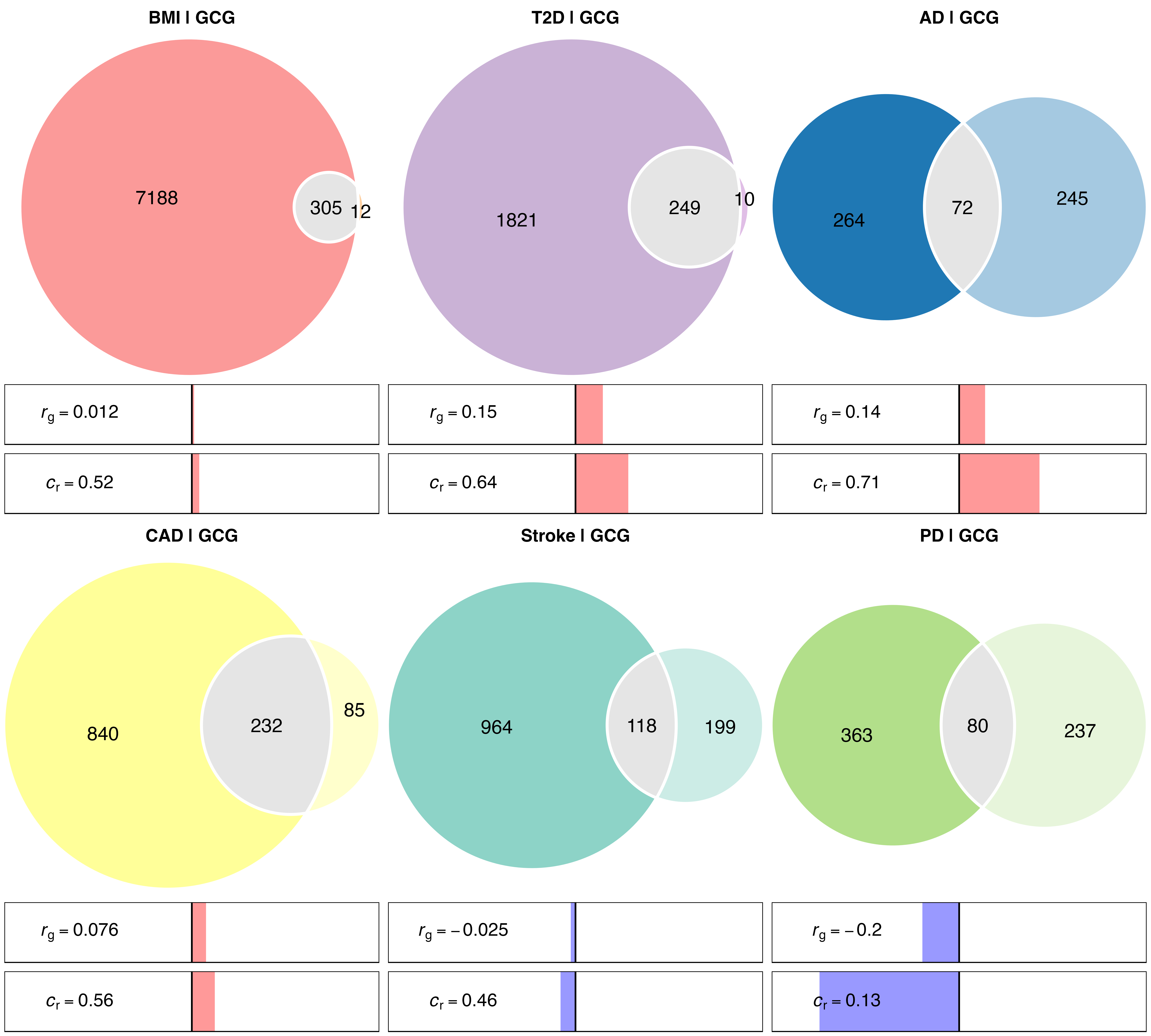


**Supplementary Fig. S7**


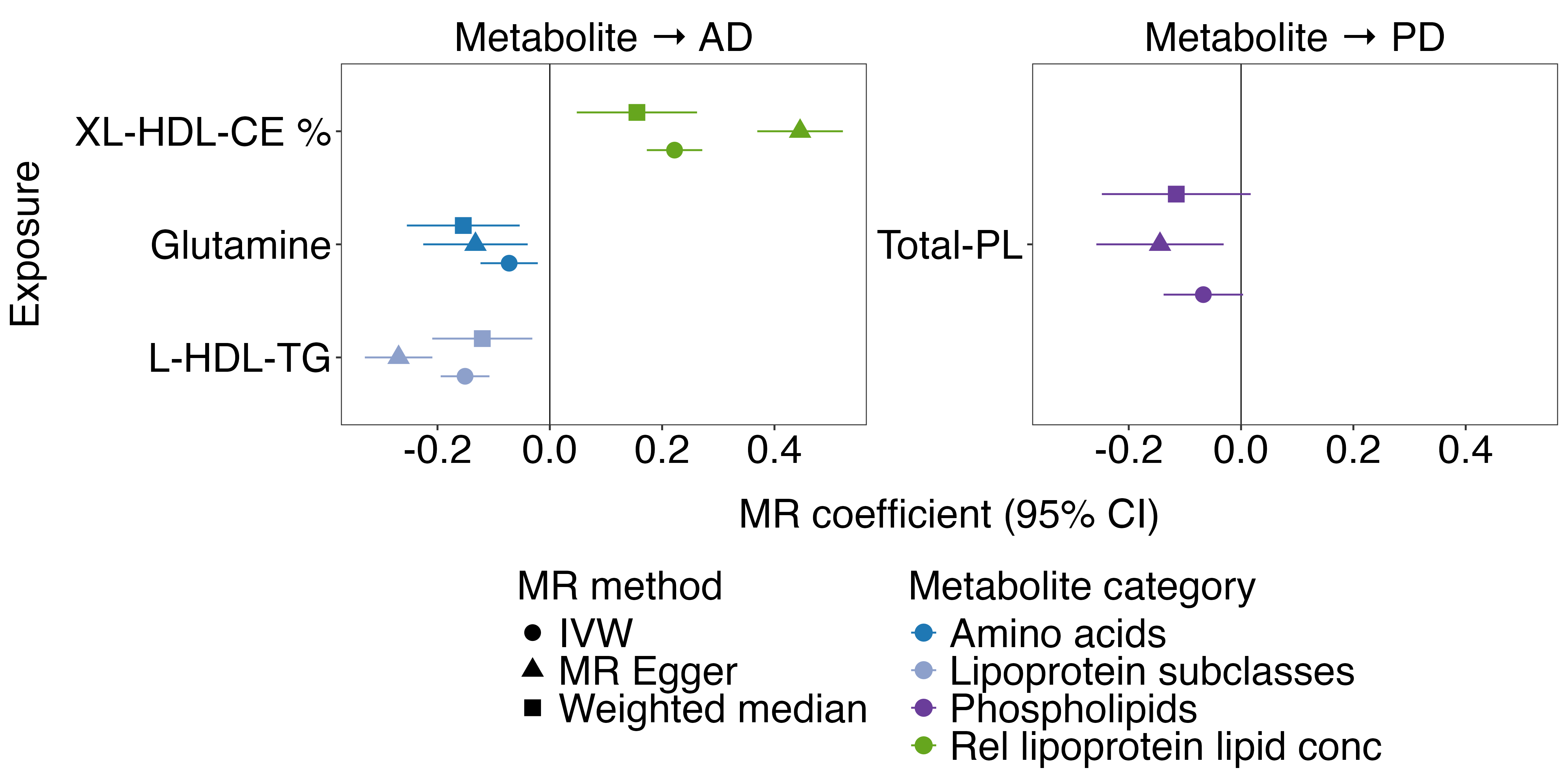


**Supplementary Fig. S8**


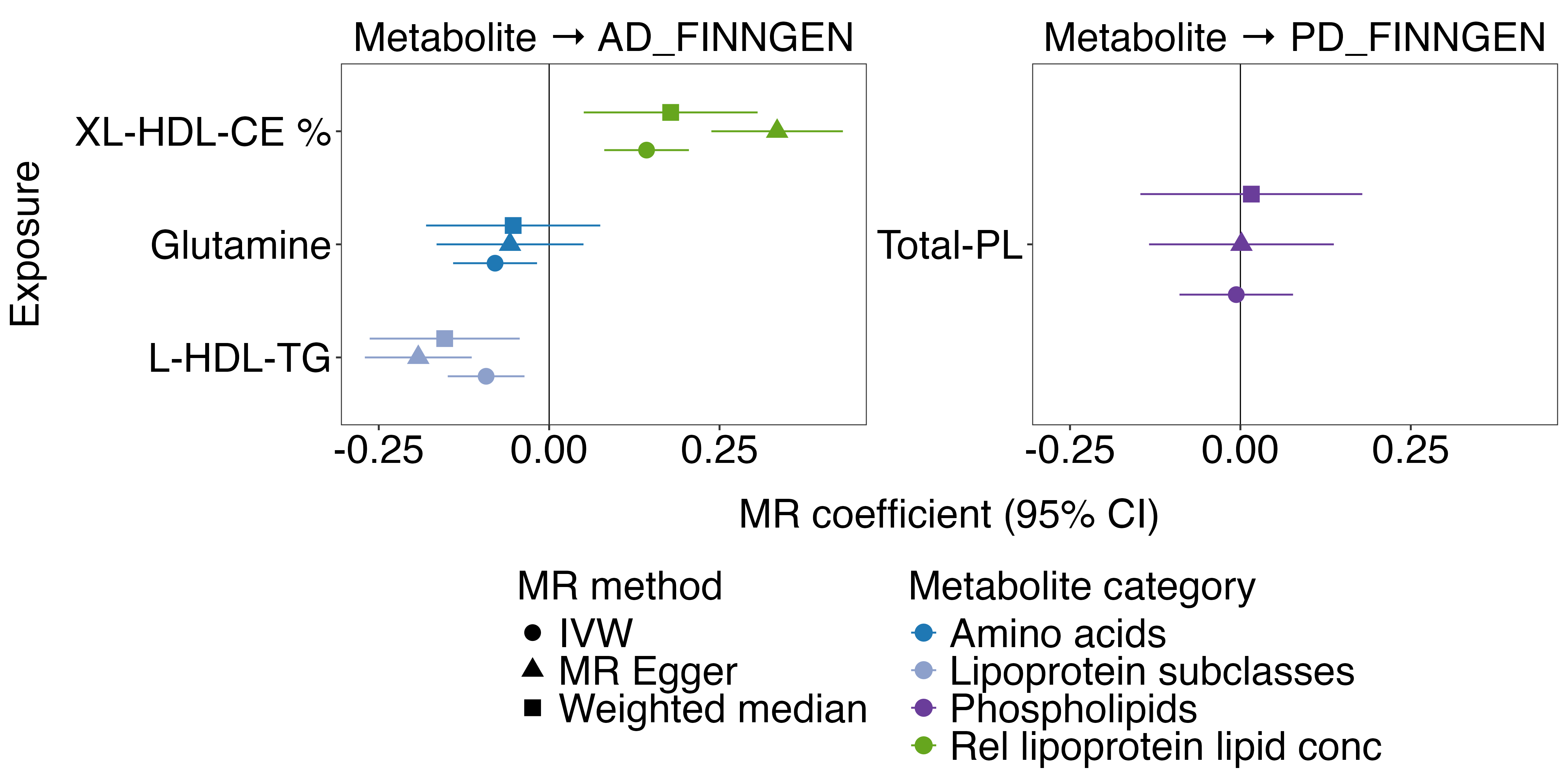


**Supplementary Fig. S9**


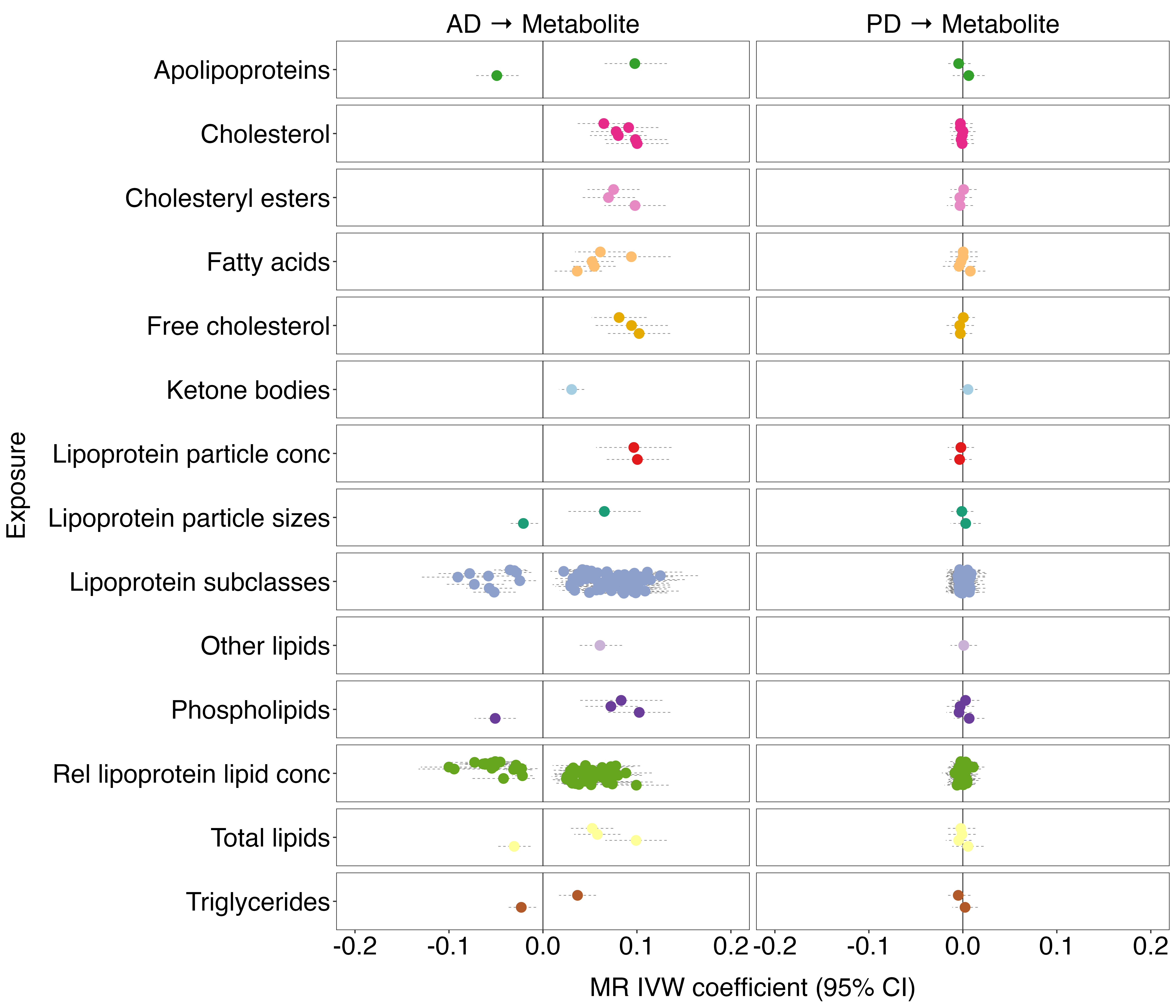


**Supplementary Fig. S10**

***
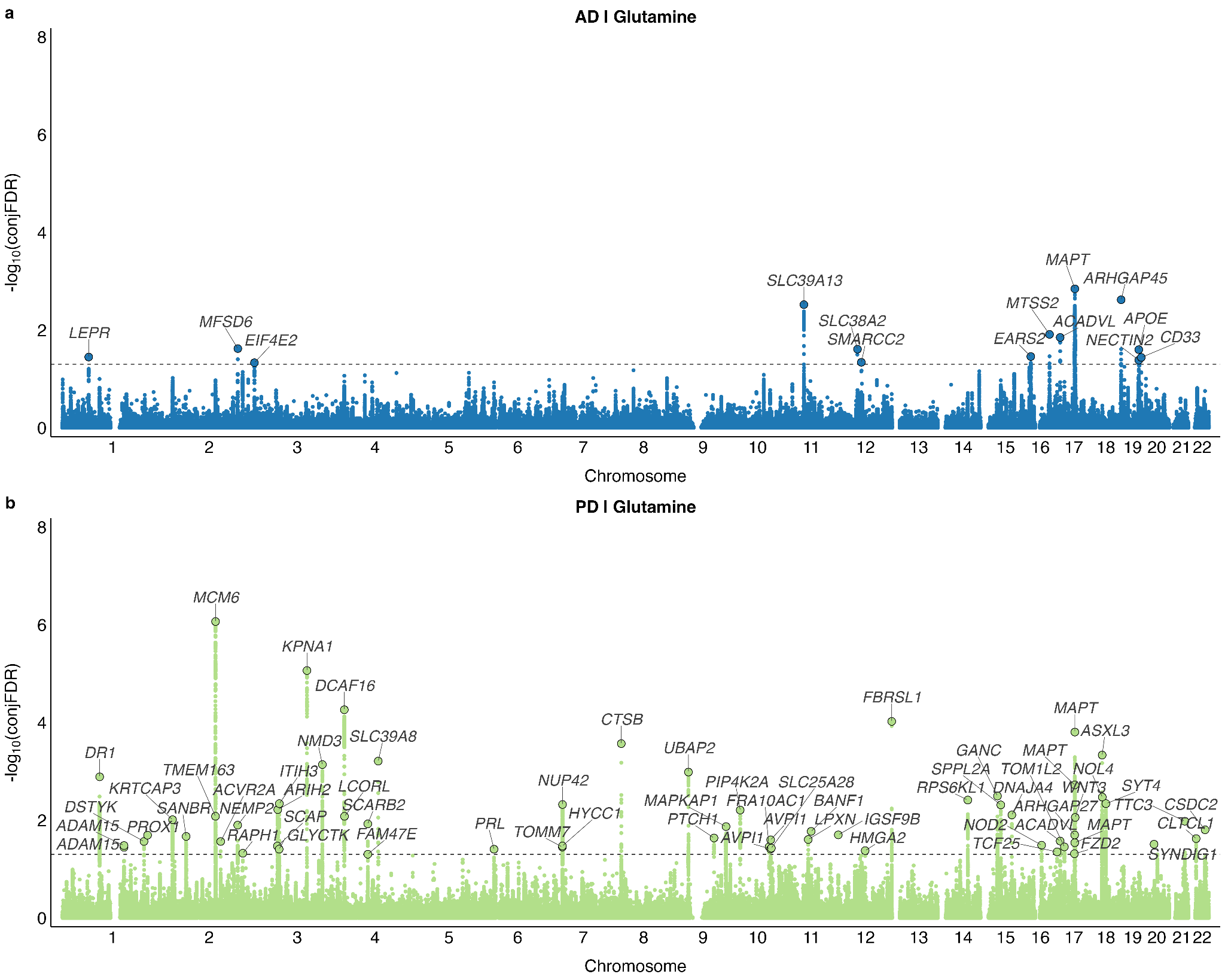
***

**Supplementary Fig. S11**

***
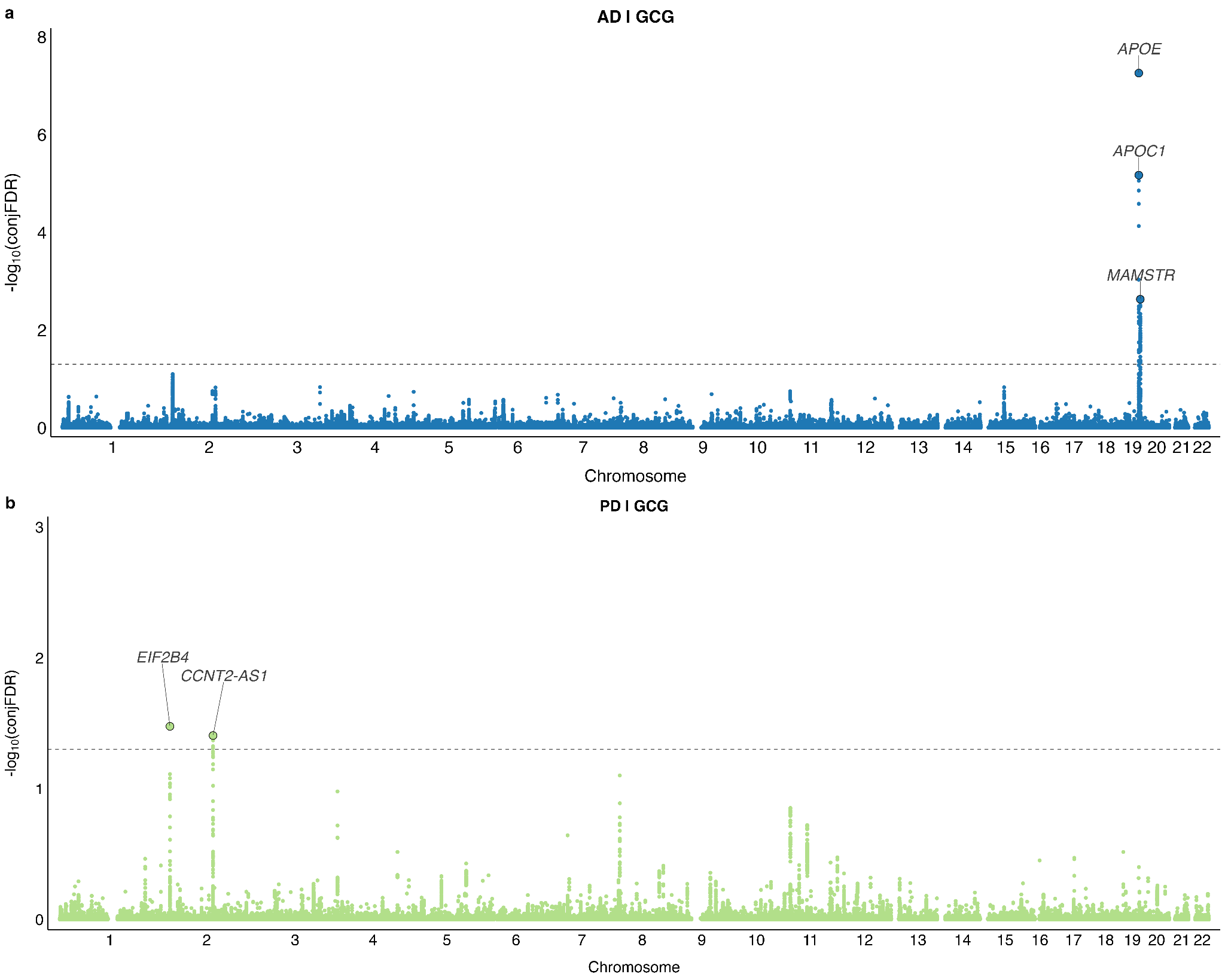
***
